# Effects of High-Intensity Swimming on Lung Inflammation and Oxidative Stress in a Murine Model of DEP-Induced Injury

**DOI:** 10.1371/journal.pone.0137273

**Published:** 2015-09-02

**Authors:** Leonardo C. M. Ávila, Thayse R. Bruggemann, Franciane Bobinski, Morgana Duarte da Silva, Regiane Carvalho Oliveira, Daniel Fernandes Martins, Leidiane Mazzardo-Martins, Marta Maria Medeiros Frescura Duarte, Luiz Felipe de Souza, Alcir Dafre, Rodolfo de Paula Vieira, Adair Roberto Soares Santos, Kelly Cattelan Bonorino, Deborah de C. Hizume Kunzler

**Affiliations:** 1 Department of Physical Therapy, State University of Santa Catarina, Florianopolis, Brazil; 2 Departments of Medicine (LIM-5 and LIM 20), School of Medicine, University of Sao Paulo, Sao Paulo, Brazil; 3 Department of Biological Science, Federal University of Santa Catarina, Florianopolis, Brazil; 4 Department of Physical Therapy, Pampa Federal University, Uruguaiana, Rio Grande do Sul, Brazil; 5 Laboratory of Experimental Neuroscience, Postgraduate Program in Health Science, University of Southern Santa Catarina at Palhoça, Santa Catarina, Brazil; 6 Health Sciences Center, Brazil Lutheran University, Santa Maria, Rio Grande do Sul, Brazil; 7 Department of Biochemistry, Federal University of Santa Catarina, Florianopolis, Brazil; 8 Postgraduate Program in Rehabilitation Sciences, Nove de Julho University, Sao Paulo (UNINOVE), Brazil; University Children's Hospital Basel, SWITZERLAND

## Abstract

Studies have reported that exposure to diesel exhaust particles (DEPs) induces lung inflammation and increases oxidative stress, and both effects are susceptible to changes via regular aerobic exercise in rehabilitation programs. However, the effects of exercise on lungs exposed to DEP after the cessation of exercise are not clear. Therefore, the aim of this study was to evaluate the effects of high-intensity swimming on lung inflammation and oxidative stress in mice exposed to DEP concomitantly and after exercise cessation. Male Swiss mice were divided into 4 groups: Control (n = 12), Swimming (30 min/day) (n = 8), DEP (3 mg/mL—10 μL/mouse) (n = 9) and DEP+Swimming (n = 8). The high-intensity swimming was characterized by an increase in blood lactate levels greater than 1 mmoL/L between 10th and 30th minutes of exercise. Twenty-four hours after the final exposure to DEP, the anesthetized mice were euthanized, and we counted the number of total and differential inflammatory cells in the bronchoalveolar fluid (BALF), measured the lung homogenate levels of IL-1β, TNF-α, IL-6, INF-ϫ, IL-10, and IL-1ra using ELISA, and measured the levels of glutathione, non-protein thiols (GSH-t and NPSH) and the antioxidant enzymes catalase and glutathione peroxidase (GPx) in the lung. Swimming sessions decreased the number of total cells (p<0.001), neutrophils and lymphocytes (p<0.001; p<0.05) in the BALF, as well as lung levels of IL-1β (p = 0.002), TNF-α (p = 0.003), IL-6 (p = 0.0001) and IFN-ϫ (p = 0.0001). However, the levels of IL-10 (p = 0.01) and IL-1ra (p = 0.0002) increased in the swimming groups compared with the control groups, as did the CAT lung levels (p = 0.0001). Simultaneously, swimming resulted in an increase in the GSH-t and NPSH lung levels in the DEP group (p = 0.0001 and p<0.002). We concluded that in this experimental model, the high-intensity swimming sessions decreased the lung inflammation and oxidative stress status during DEP-induced lung inflammation in mice.

## Introduction

Nowadays air contamination is a real problem of public health in urban areas, and motor vehicle emissions, undoubtedly, are a major source of airbone pollutants. Epidemiological studies showed a strong correlation between the air concentration of pollutants and the increase in mortality, hospitalizations and emergency care, mostly related to the aggravation of established respiratory and heart diseases. In healthy individuals, new cases of these diseases were associated with levels of air pollution [1–4].

One of the classic pollutants composing the biomass in the air is particulate matter (PM). This term describes a mixture of solid or liquid particles dispersed in the air; the exhaust from vehicles and industries is the main source of PM [5,6]. The particles that result from burning diesel are essentially composed of metals, polycyclic aromatic hydrocarbons, and other organic species are called diesel exhaust particles (DEP), and affect different aspects of human health and disease [7–11]. The component of DEP responsible for the oxidative stress and subsequent pro-inflammatory signaling is especially the organic fraction, although transition metals may also be involved [7,8].

The oxidative stress triggered by DEP causes activation of signaling pathways such as those involving NF-κB and histone acetylation favoring pro-inflammatory gene expression. Interleukin-8 (IL-8) was induced in epithelial cells treated *in vitro* and in human lungs exposed by inhalation [12]. Tumor necrosis factor-alpha (TNF-α) has been reported to be increased in macrophages exposed to DEP *in vitro* and interleukin-6 (IL-6) is released by primed human bronchial epithelial cells exposed to DEP [12–14].

The respiratory tract is particularly affected because of its huge internal surface and the fact that it is one of the first sites to be exposed to air pollution. The inhalation and deposition of DEPs result in cell activation and the release of many pro-inflammatory molecules. Inflammatory processes are well-known contributors to the development and exacerbation of lung diseases, such as asthma, chronic obstructive pulmonary disease (COPD), acute respiratory distress syndrome (ARDS), and lung cancer [15–17].

Previous experimental studies have shown that short- and long-term administration of DEPs may activate immune cells, including neutrophils and macrophages; thus, DEPs contribute to the release of inflammatory cytokines in the lungs, such as interleukin 1β (IL-1β), interleukin 6 (IL-6), chemokine ligand 1 (CXCL-1/KC) and tumor necrosis factor α (TNF-α) [5,6,9,11]. One of the possible mechanisms for the release of pro-inflammatory cytokines is related to the production of reactive oxygen (ROS) and nitrogen species (RNS) that activate transcription factors such as nuclear factor kappa β (NF-kβ), disrupting the oxidant/antioxidant balance [9,18,19]. Furthermore, the increased oxidative stress status caused by activated immune cells may also contribute to the resulting DNA damage [20,21].

Aerobic exercise is a powerful tool to combat oxidative stress activation; it also provides a protective mechanism that helps to re-establish cellular homeostasis, decrease the release of pro-inflammatory cytokines and production of ROS, and improve immune responses [22–24].

Several studies, including clinical trials, have shown that aerobic exercise produces anti-inflammatory and antioxidant effects in different diseases, such as heart disease [25], diabetes [26], Alzheimer’s disease [27], and Parkinson’s disease [28], as well as in lung diseases, such as COPD [29–31], ARDS [32,33] and asthma [34–38]. Notably, Vieira et al., (2011) showed that aerobic exercise at low and moderate intensities resulted in the immunomodulation of epithelial cells from the airway of animals with long-term allergic lung inflammation, thus decreasing the oxidative stress and lung inflammation [36]. In addition, in a murine experimental model, the same authors also showed that when low-intensity exercise is performed simultaneously with DEP administration, there are decreases in pro-inflammatory cytokine release and pulmonary oxidative stress, as well as an inhibition of the lung and systemic inflammation [39].

However, the literature still contains controversial results concerning the effects of high-intensity aerobic exercise on health and disease. Hall et al. (2013) showed that high-intensity aerobic exercise resulted in the greatest reduction in insulin dosage compared with that for low- or moderate-intensity treadmill training [40]. Furthermore, Balducci et al. (2010) also showed that high-intensity aerobic exercise improves the inflammatory status in diabetic patients [41]. However, Camiletti-Moirón et al. (2013) demonstrated a deteriorated antioxidant response in the brains of rats trained using high-intensity aerobic exercise [42]. Regarding lung effects, specifically, Stang et al. (2014), showed that after high-intensity exercise, there is a significant reduction in exhaled nitric oxide concentration, known by its inflammatory role into the lungs [31]. These studies highlighted the anti-inflammatory and pro-inflammatory response, as well as the changes in redox balance, induced by high-intensity aerobic exercise [40–43].

Due to its dual feature and influence on redox balance, we hypothesized that high-intensity swimming could decrease inflammatory markers by decreasing oxidative stress and increasing antioxidants defenses into lungs [30,31]. Thus, in this study a short period high-intensity exercise (swimming) maintained blood lactate at elevated levels between the 10^th^ and 30^th^ minute of exercise. This, contrasts with long term exercise protocols, in which an adaptation to exercise keeps blood lactate levels lower than 1 mmoL/L [43].

Therefore, the present study aimed to evaluate the effects of high-intensity swimming, which was performed prior to and simultaneously with DEP administration, on lung inflammatory and oxidative stress responses.

## Materials and Methods

This study was approved by the Review Board for Animal Studies of the Federal University of Santa Catarina. All animals in the study received humane care in compliance with EU Directive 2010/63/EU, which provides guidelines for animal experiments [44].

### 2.1 Collection, analysis and suspension of diesel exhaust particles (DEP)

A particle trap device was adapted for use with the exhaust pipe of a bus from the public transportation fleet of São Paulo city. The bus was equipped with a Mercedes Benz MB1620 210-hp engine, lacked electronic control of fuel injection, and was fueled with diesel containing 500 ppm sulfur. This particular type of bus was chosen because it is the most frequently operated bus in São Paulo, according to information provided by the municipality. Briefly, a mesh made of stainless steel was inserted into the exhaust pipe line of the bus. Diesel particles were collected during 1 day of routine operation of the bus, and these particles were stored for toxicological studies. Twenty-four hours after the collection, the composition of the particles was analyzed [45]. These DEP samples were dispersed in 0.9% NaCl for 8 hours prior to use in the experiments [45]. The experimental DEP concentration (3 mg/mL) and protocol of instillation were based on a previous work of our group [39], showing that DEP instillation decreases exercise capacity and induces oxidative stress markers and pro-inflamatory cytokynes.

### 2.2 Animals and experimental groups

Thirty-seven male Swiss mice (30–35 g) from the Animal Facility of Federal University of Santa Catarina were maintained in conditions that were controlled for temperature (22 ± 2°C), humidity (70–75%) and dark/light cycle (12 h; lights on at 06:00 am). The experimental protocol was initially set with 12 mice in each group (total of 48 animals). Some animals get injuried during experimental protocol (n = 4 in Swimming group, n = 3 in DEP group and n = 4 in DEP+Swimming group) and were removed from the experiment.

Thus, animals were assigned to 4 groups as follows: a) Control group (mice that were not subjected to swimming and received intranasal saline instillation during 10 days, n = 12); b) Swimming group (Sw): animals that were forced to swim (30 minutes/day) from 1^st^ to the 10^th^ experimental day, and received intranasal saline instillation (n = 8); c) DEP group (mice that were not subjected to swimming and received DEP instillation during 10 days, n = 9) and, d) DEP+Swimming group (DEP+Sw): mice that were subjected to swimming and received DEP instillation, n = 8).

### 2.3 DEP administration and swimming protocol

DEPs (3 mg/mL) were intra-nasally administered (10 μL/mouse) using a 100 μL micropipette. The intra-nasal administration induces reflex apnea, followed by a deep inspiration, which drives the solution into the lungs. The saline-administered groups received vehicle (0.9% NaCl).

**Fig 1:**
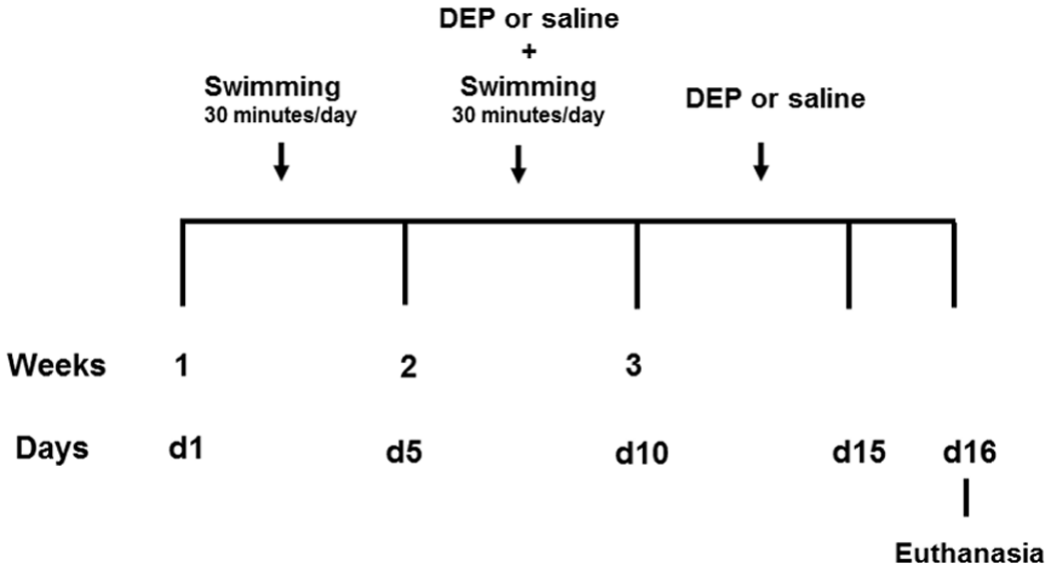
Time line of the experimental protocol. Swimming was initiated on day 1, and ended on the 10^th^ experiment day. DEP instillation occurred from 5^th^ to 10^th^ day of the swimming protocol, and until the 15^th^ day, the animals were submitted only to daily DEP or saline instillation. Animals were euthanized 24 hours after the last instillation.

The animals were put to swimming on a daily basis, according to experimental protocol shown at Fig 1. DEP particles were administered just before swimming, without any kind of animal narcosis.

The swimming protocol was adapted from Kuphal, Fibuch and Taylor (2007) [46]. A box containing 35 L of warm water (kept at 35°C during the exercise) was divided into 8 lanes, and we added 1 mL of shampoo (Johnson & Johnson) to reduce the surface tension and prevent floating. All groups were submitted to the same adaptation procedure: animals were initially adapted to the water environment for 4 days: day 1, two periods of 30 seconds of swimming with an interval of 2 hours between the swimming periods; day 2, two periods of 2 minutes of swimming separated by an interval of 2 hours; day 3, three periods of 10 minutes of swimming separated by intervals of 5 minutes, and day 4, two periods of 15 minutes of swimming separated by intervals of 5 minutes. After the adaptive period, the mice were subjected to swimming for 30 minutes with no pause from day 1 to day 10.

### 2.4 Blood lactate measurements

The intensity of the exercise was determined using an adapted protocol [47]. The blood lactate concentration in the Swimming and DEP+Swimming groups was measured at the 5^th^ and 10^th^ days of exercise at the 10^th^ and 30^th^ minutes of swimming. At the 5^th^ experimental day, the Control and DEP groups were also subjected to 30 min exercise in order to obtain the lactate concentration, which was also measured at the 10^th^ and 30^th^ minute of swimming.

### 2.5 Bronchoalveolar lavage fluid (BALF)

Twenty-four hours after the final DEP administration, the animals were anesthetized with ketamine (10 mg/kg i.p) and xylazine (10 mg/kg i.p), and a tracheotomy was performed for BALF collection. Before the BALF collection, the mice were euthanized by rapid exsanguination by sectioning the abdominal aorta. The lungs were gently rinsed 3 times with 0.5 mL of phosphate buffered saline (PBS, pH 7.2) via a tracheal cannula. The total cell number was counted in a Neubauer hemocytometer chamber. The differential cell count of 300 cells/mouse was obtained after Diff-Quick staining of slides prepared with the BALF. All measurements were performed in a blinded fashion.

### 2.6 Analysis of cytokines

After the BALF collection, the chest was opened, and the heart-lung block was removed. The left lung was separated, collected in Eppendorf tubes and homogenized in a tissue processor (Ultra-Turrax IKA T18 basic, IKA, Germany) with a phosphate-buffered saline (PBS) solution containing the following: Tween 20 (0.05%), 0.1 mM phenylmethylsulphonyl fluoride (PMSF), 10 mM ethylenediaminetetraacetic acid (EDTA), 2 ng/ml Aprotinin and 0.1 mM benzethonium chloride. The homogenates were transferred to 1.5 mL Eppendorf tubes and centrifuged at 3000 ×*g* for 10 min at 4°C, and the resulting supernatant was stored at -80°C until cytokine analysis. The total protein content in the supernatant was measured using the Bradford method. The lung tissue levels of interleukin 10 (IL-10), interleukin 1ra (IL-1ra), interleukin 1β (IL-1β) tumor necrosis factor alpha (TNF-α), interleukin 6 (IL-6) and interferon gamma (IFN-γ) were measured using the ELISA technique according to the manufacturer’s instructions (DuoSet ELISA R&D Systems, Minneapolis, MN, USA). The cytokine levels were estimated by interpolation from a standard curve using colorimetric measurements at 450 nm (correction wavelength 540 nm) in an ELISA plate reader (Berthold Technologies—Apollo 8 –LB 912, KG, Germany). All results were expressed as pg/mg of protein.

### 2.7 Analysis of antioxidant defenses

Samples of fresh lung tissue (right lung) weighing approximately 40–50 mg were used for the analysis of the levels of total GSH (glutathione) and NPSH (non-protein thiols). The activities of glutathione peroxidase (GPx) and catalase (CAT) were measured using frozen lung tissue homogenate. The non-protein thiols were measured by the spontaneous reaction of Ellman's reagent (5,5 'dithiobis-2-nitrobenzoic; DTNB) with sulfhydryl groups to produce 5-thio-2-nitrobenzoic acid (TNB), which absorbs light at 412 nm [48]. An aliquot of the supernatant obtained from the centrifugation of the samples was added to TRIS/HCl 2.5 M pH 8 and 0.2 mM DTNB. The samples were then measured spectrophotometrically at 412 nm. The total glutathione was measured using the Tietze method modified by Akerboom and Sies (1981) [49]. The method also relies on the spontaneous reaction of DTNB with GSH to form TNB and GSTNB. The GSTNB is reduced back to GSH by the enzyme glutathione reductase, which uses NADPH as an electron donor and releases TNB. The rate of formation of TNB was measured spectrophotometrically at 412 nm for 2 minutes and compared to a standard curve.

### 2.8 Statistical analysis

Comparisons among groups were performed using Sigma Stat 3.5 software (California, EUA, 2005) by Two-Way analysis of variance (ANOVA) followed by Holm-Sidak test for multiple comparisons. Data showed normal distribution, as analyzed by the Kolmogorov-Smirnov test. Significance levels were set at 5% (p <0.05). Values were expressed as the mean ± standard deviation (SD).

## Results

### 3.1 Swimming sessions increased the blood lactate concentration

The swimming sessions resulted in an increase in the blood lactate concentration between the 10^th^ and 30^th^ minute of exercise (Table 1), which was measured on each of the 5 days of the exercise protocol. These data confirm that this protocol can be considered a model of high-intensity exercise. As can be seen in Table 1, all groups subjected to the swimming sessions presented blood lactate concentration higher than 1 mmol/L.

**Table 1:** Blood lactate concentration. Table 1 shows the values (mean ± SD) of blood lactate concentration, expressed in mmol/L. Control and DEP groups were submitted only once to exercise protocol, in order to establish lactate blood basal control.

Time	Control	Sw	DEP	DEP + Sw
10° min	3.5± 1.1	4.1± 0.7	4.4± 1.4	3.7± 1.6
30° min	5.6± 1.4*	5.9± 0.3*	6.2± 1.2*	5.7± 0.8*

* = Significantly different from the 10^th^ minute of the respective group (p<0.05).

### 3.2 Swimming sessions decreased the number of BALF cells

**Fig 2:**
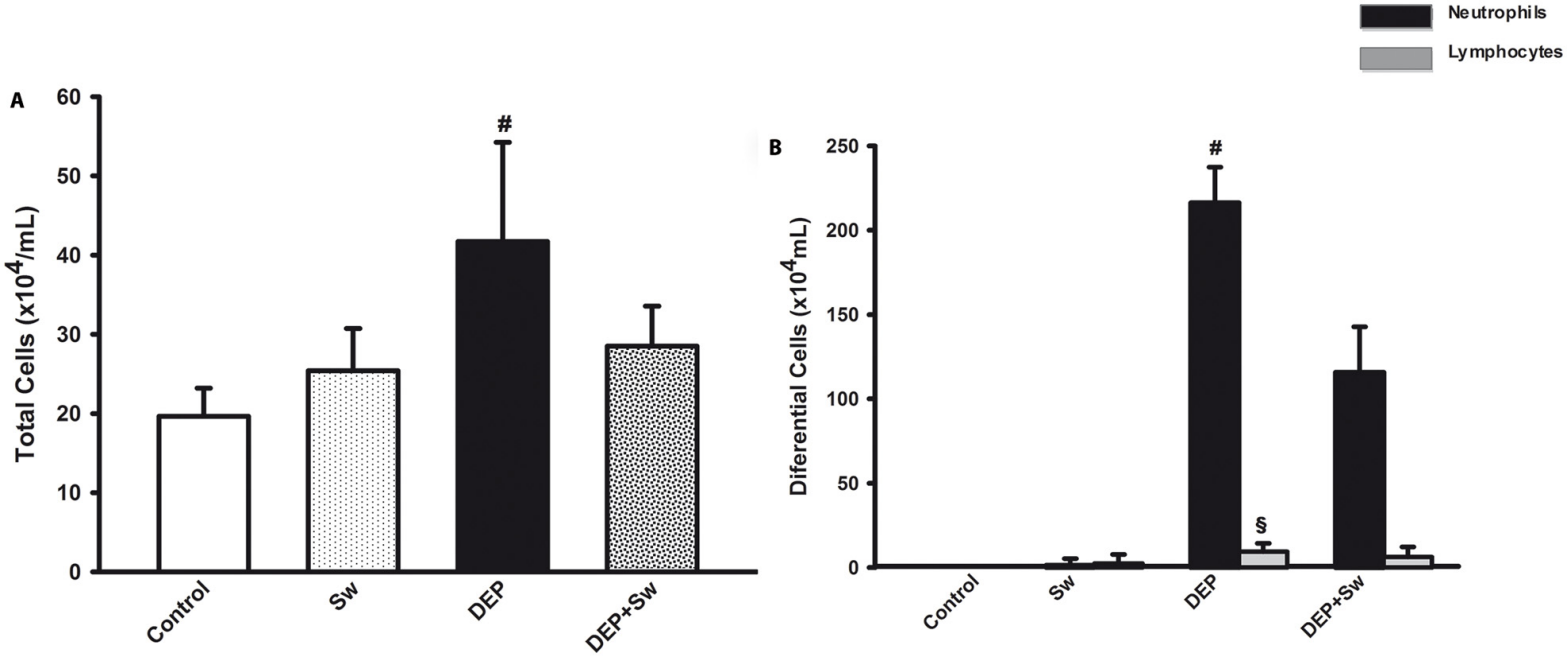
Cells from BALF. Fig 2A shows the total number of cells from BALF. Values are expressed as mean ± SD. ^#^p<0.001 when compared to all groups. Fig 2B shows the differential counting of neutrophils and lymphocytes from BALF. ^#^p<0.001 for neutrophils when compared to all groups and ^§^p<0.05 for lymphocytes when compared to Control group.

Swimming attenuated the increase in the number of total cell and neutrophils (p<0.001). Total number of cells (Fig 2A), neutrophils, and lymphocytes (Fig 2B) were not affected by exercise, while DEP produced a marked increase in these cells. A two-way ANOVA for the total number of cells did not show any significant effect for swimming (F(3,7) = 2,68; p = 0.1); however, the effect of DEP instillation was significant (F(3,8) = 30.28; p<0.001), and swimming x DEP interaction (F(3,7) = 17.15; p<0.001) was significant. The two-way ANOVA for the neutrophils also showed significant effect for swimming (F(3,7) = 56.76; p<0.001). The effect of DEP instillation was significant (F(3,8) = 631.71; p<0.001), and swimming x DEP interaction was also significant (F(3,7) = 59.95; p<0.001). The two-way ANOVA for the lymphocytes did not show any significant effect for swimming (F(3,7) = 0.03; p = 0.8); however, the effect of DEP instillation was significant (F(3,8) = 10.03; p<0.01), and swimming x DEP interaction was not significant (F(3,7) = 1.78; p = 0.2).

### 3.3 Swimming sessions prevented the induction of pro-inflammatory cytokines in the lung

**Fig 3:**
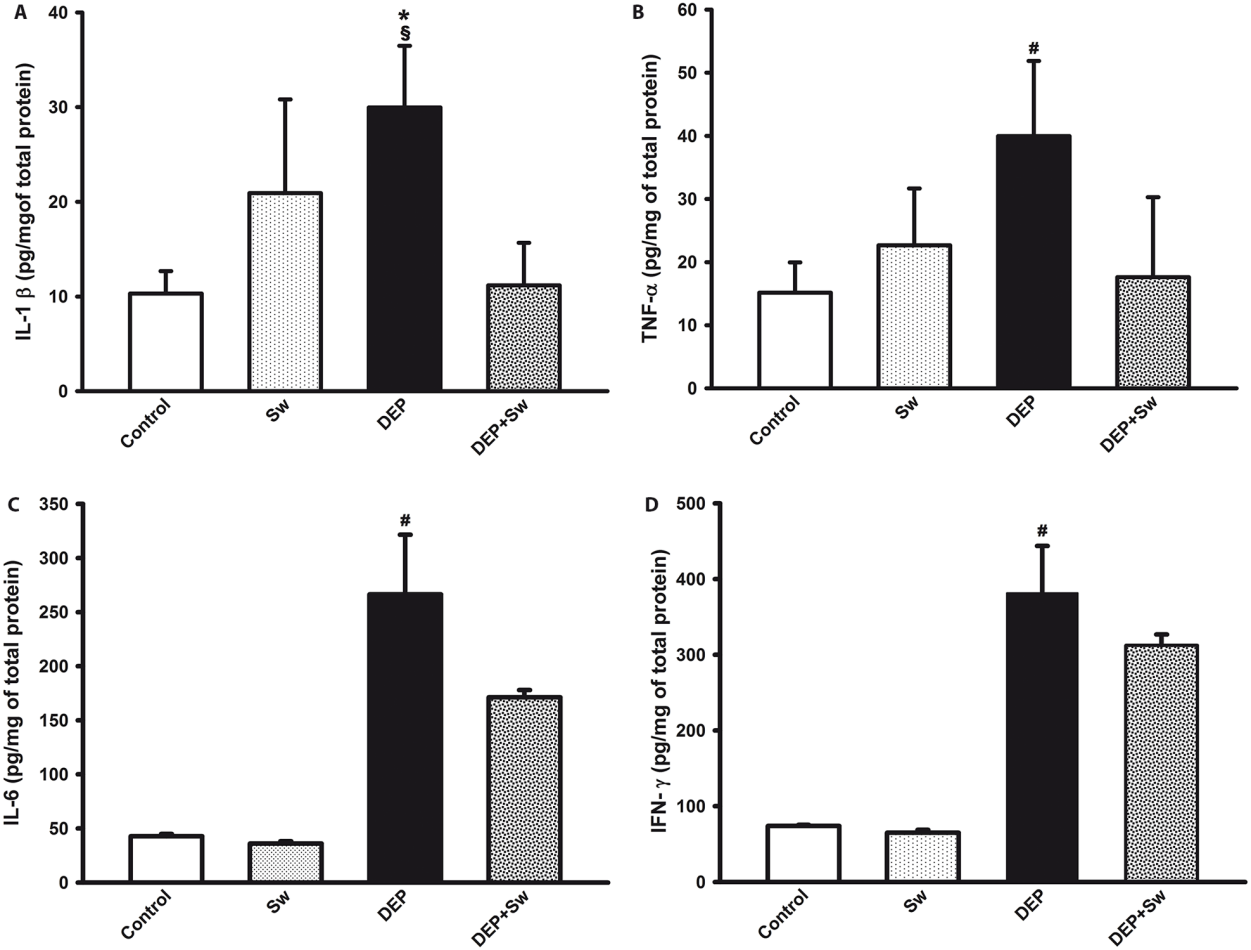
Pro-inflammatory cytokines lung levels. Fig 3 shows the total levels of IL-1 β (Fig 3A), TNF-α (Fig 3B), IL-6 (Fig 3C), IFN-γ (Fig 3D) from homogenate lung tissue. Values are expressed as mean ± SD. Fig 3A, ^§^*p = 0.002 when compared to Control and Dep+Sw groups. Fig 3B, ^§^*p = 0.003 when compared to Control and Dep+Sw groups. Fig 3C, ^#^p = 0.0001 when compared to all groups and Fig 3D, ^#^p = 0.0001 when compared to all groups also.

Swimming attenuated the increase in lung levels of IL-1β (Fig 3A) and TNF-α (Fig 3B) (p = 0.002 and p = 0.003, respectively). A two-way ANOVA did not show any significant effect on IL-1 β for swimming (F(3,8) = 2.78; p = 0.11) or DEP effect (F(3,8) = 3.68; p = 0.07. A two-way ANOVA also did not show any significant effect on TNF-α for swimming (F(3,8) = 2.20; p = 0.16) or DEP effect(F(3,8) = 3.90; p = 0.07). However, swimming x DEP interaction was significant in both cytokines lung levels (F(3,7) = 14.15; p<0.01; F(3,8) = 8.93; p = 0.01, respectively).

Similarly, swimming attenuated the increase in lung levels of IL-6 (p = 0.0001) (Fig 3C) and IFN-ɣ (p = 0.0001) (Fig 3D). Interestingly, two way ANOVA showed significant effect for swimming (F(3,8) = 27.85; p<0.001) and DEP effect (F(3,8) = 344.58; p<0.001) in IL-6 lung levels (Fig 3C), as well as swimming x DEP interaction was also significant (F(3,8) = 20.95, p<0.001). Two way ANOVA also showed significant effect for swimming (F(3,8) = 12.71; p = 0.001), DEP (F(3,8) = 637.02; p<0.001) and swimming x DEP interaction (F(3,8) = 7.59; p = 0.01) for IFN-ɣ lung levels.

### 3.4 Effects of swimming sessions on the lung levels of anti-inflammatory cytokines

**Fig 4:**
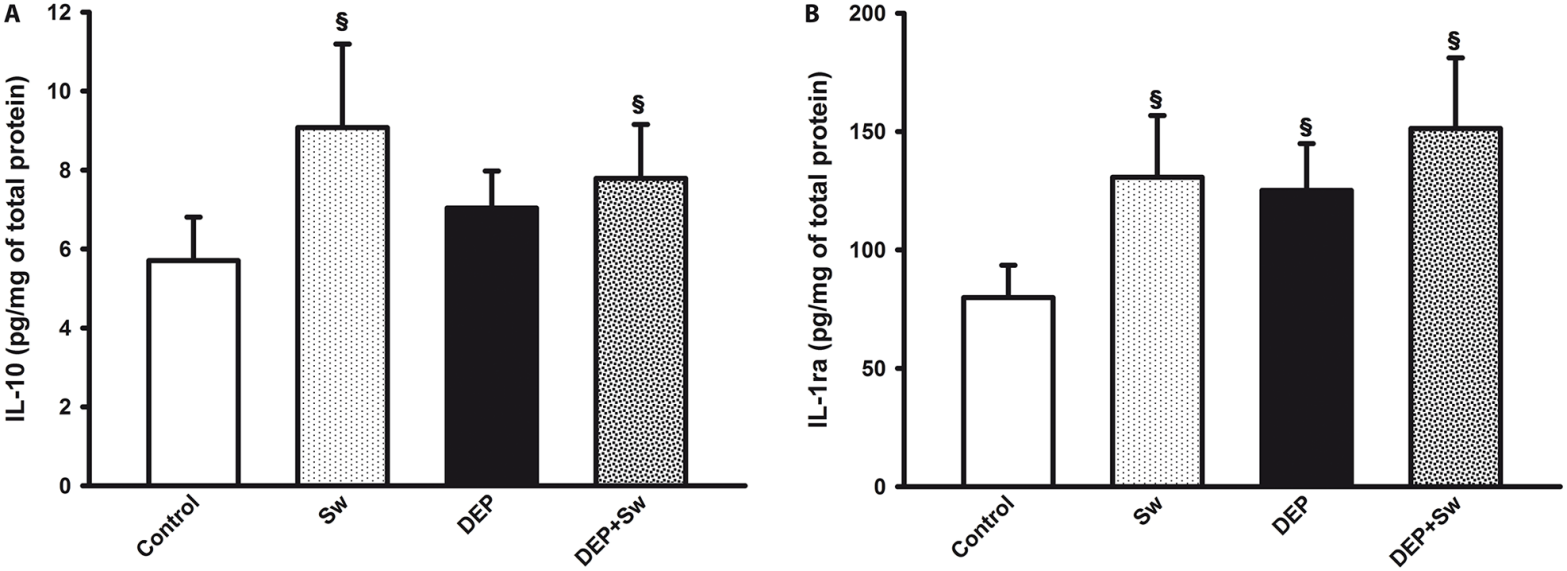
Anti-inflammatory cytokines lung levels. Fig 4 shows the total levels of IL-10 and IL-1ra (Fig 4A and 4B, respectively) from homogenate lung tissue. Values are expressed as mean ± SD. Fig 4A, ^§^p = 0.01, when compared to Control group, and Fig 4B, ^§^p = 0.0002, when compared to Control group also. When compared DEP and DEP+Sw groups, there was no significant statistical difference (p>0.05) in both measurements.

Swimming sessions resulted in an increase in the levels of anti-inflammatory cytokines (p = 0.01 for IL-10, Fig 4A) (p = 0.0002 for IL-1ra, Fig 4B). A two way ANOVA for lung levels of IL-10 (Fig 4A) showed significant effect for swimming (F(3,7) = 8.90; p = 0.01), but did not show significance for DEP effect (F(3,7) = 0.001; p = 0.97) and for swimming X DEP interaction (F(3,7) = 3.60; p = 0.08).

Two way ANOVA also revealed that levels of IL-1ra (Fig 4B) showed significant effect for swimming (F(3,7) = 17.99; p<0.001) and DEP effect (F(3,8) = 13.233; p = 0.01), but did not show significance for swimming X DEP interaction (F(3,7) = 1.85; p = 0.185).

### 3.5 Swimming sessions increased the CAT lung level

**Fig 5:**
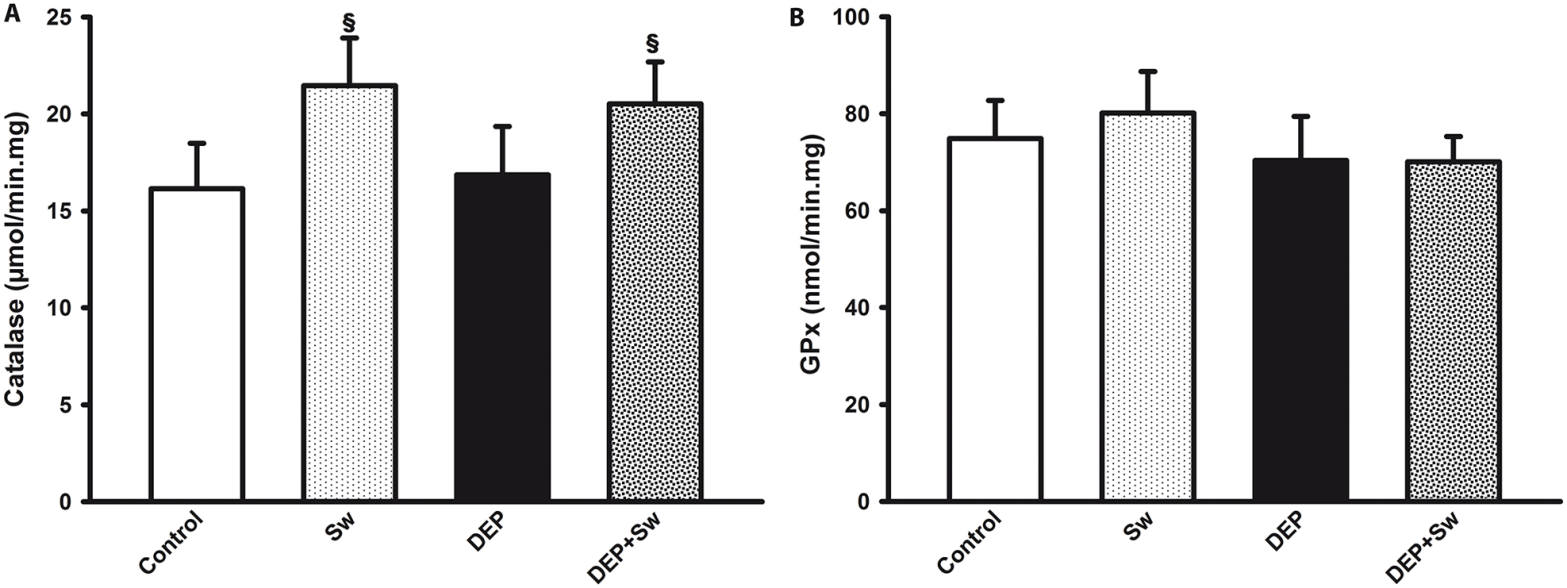
Antioxidant enzymes lung levels. Fig 5 shows the pulmonary levels of CAT and GPx (Fig 5A and 5B, respectively). Values are expressed as mean ± SD. Fig 5A, ^§^p = 0.0001, when compared to Control group.

The lung level of CAT (Fig 5A) was not affected by DEP instillation, while high-intensity swimming produced a marked increase in this anti-oxidant enzyme into lungs (p = 0.0001). A two way ANOVA for lung levels of CAT showed significant effect for swimming (F(3,7) = 21.15; p<0.001), but did not show any significant DEP effect (F(3,8) = 0.03; p = 0.8) and for swimming x DEP interaction (F(3,7) = 0.9; p = 0.3). Additionally, two way ANOVA also showed that there were no significant differences in GPx lung levels (Fig 5B) for swimming training F(3,7) = 0.5; p = 0.4) and swimming x DEP interaction (F(3,7) = 0.77; p = 0.38), although there is a significant DEP effect (F(3,8) = 6.42; p = 0.01).

### 3.6 DEP exposure decreases the lung GSH and NPSH levels

**Fig 6:**
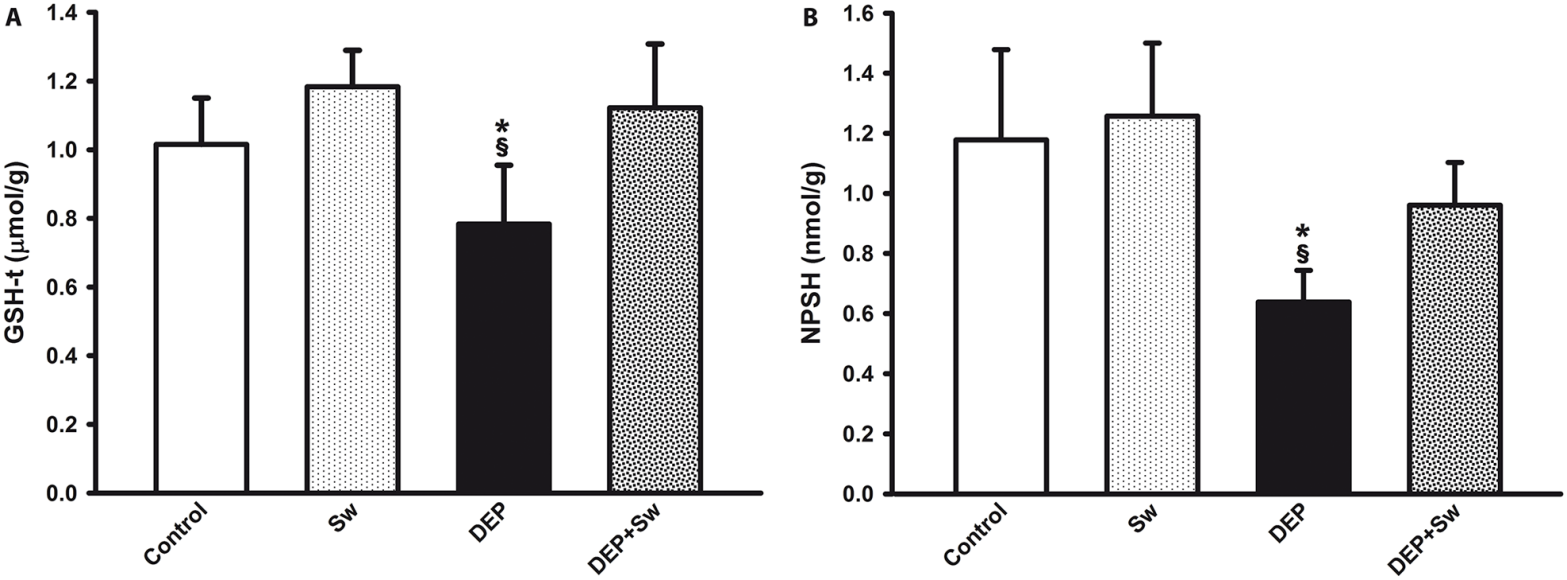
Thiols lung levels. Fig 6 shows the lung levels of GSH-t and NPSH (Fig 6A and 6B, respectively). Values are expressed as mean ± SD. Fig 6A and 6B, ^§^*p = 0.0001 and ^§^*p = 0.002, respectively, both when compared to Control and DEP+Sw groups.

DEP instillation resulted in a pronounced decrease of GHS and NPSH lung levels (Fig 6A and 6B, respectively), while swimming sessions avoided a decrease in thiols lung levels (p = 0.0001 for GSH and p = 0.002 for NPSH. A two way ANOVA for GSH lung levels showed significant differences for swimming (F(3,7) = 25.93; p<0.001), but not for DEP (F(3,8) = 3.12; p = 0.08). However, the swimming x DEP interaction was significant (F(3,7) = 10.82; p = 0.003). In addition, DEP instillation also resulted in a decrease of NPSH lung levels (Fig 6B). A two way ANOVA for NPSH lung levels showed significant effect for swimming (F(3,7) = 4.96; p = 0.03), as well as a significant DEP effect (F(3,8) = 21.55; p<0.001), but did not show swimming x DEP interaction (F(3,7) = 1.84; p = 0.19).

## Discussion

The present study provides the first demonstration that mice subjected to high-intensity swimming sessions and DEP administration for 10 days showed a decrease in the number of total cells, neutrophils and lymphocytes in BALF, even when the exercise was interrupted and the DEP administration was continued for 5 more days. Furthermore, exercise also decreased the levels of pro-inflammatory cytokines, such as IL-1β, IL-6, TNF-α and IFN-ϫ, in the lung homogenates, whereas exercises increased the levels of anti-inflammatory cytokines, such as IL-1ra and IL-10. In addition, swimming resulted in an increase in the levels of glutathione, non-protein thiols and catalase, which are known antioxidant molecules, in the lung homogenates.

Diesel particles from the atmosphere (DEPs) are the largest source of the particulate matter that is associated with various deleterious effects on human health [7–14,45]. In epidemiological studies and experimental models, exposure to DEPs has been identified as a potent source of immunomodulation [50,51]. Salvi et al. (1999) observed the effects of acute exposure to DEPs in the blood and BALF of volunteer subjects exposed to DEPs in a controlled exposure chamber. After 1 hour of DEP exposure, the authors observed a significant increase in the number of mast cells, neutrophils, B lymphocytes, and the CD4 and CD8 subtypes of T lymphocytes, as well as increased IL-8 levels and an increase in the expression of ICAM-1 and VCAM [52].

Results from experimental models also have confirmed the impairment caused by DEP exposure, showing the increase in reactive oxygen and nitrogen species, either systemically or locally in different organs [9,18,19]. Additionally, some studies suggest that DEP particles engulfed by immune and epithelial cells may induce an early hypersensitive response and subsequent DNA damage triggered by the break down and release of the soluble chemical DEP components in cells, which induces pulmonary inflammation and redox imbalance [10–14,39].

However, animal and clinical studies have suggested that aerobic exercise has a protective effect against DEP exposure, decreasing the release of inflammatory mediators and reactive oxygen species (ROS). Vieira et al. (2012) demonstrated that low-intensity aerobic exercise resulted in a decreased release of pro-inflammatory cytokines and decreased levels of oxidative and nitrosative stress, as well as inhibition of both lung and systemic inflammation; these results suggested a possible modulation of the beneficial effects of exercise through anti-inflammatory and antioxidant pathways [39]. This theory could be supported by other studies showing that the beneficial effects of exercise may be directly related to the anti-inflammatory and antioxidant response induced by exercise [22–24,53].

In our study, we showed that DEP exposure resulted in a significant increase in the number of BALF cells (total cells, neutrophils and lymphocytes), and swimming sessions decreased the number of total cells, neutrophils and lymphocytes. These data corroborate those of Vieira et al. (2012), who demonstrated a decrease in inflammatory cells not only in the BALF but also in the lung parenchyma of mice exposed to DEPs and subjected to exercise [36]. When activated by chemical substances (e.g., the substances in DEP), inflammatory cells (e.g., macrophages and neutrophils) secrete ROS [30,39]. In our study, we demonstrated that swimming sessions were able to significantly improve the antioxidant defense, as demonstrated by improvements in the levels of CAT, GSH-t and NPSH. These findings could then partially explain the antioxidant effects of swimming in this model of DEP-induced lung inflammation. Similarly, Vieira et al. (2012) have shown that aerobic exercise reduces ROS and RNS in a model of DEP-induced lung inflammation [39].

Any acute condition of increased oxygen consumption leads to oxidative stress and consequently ROS production. Thus, the repeated mild increases in ROS caused by exercise results in an adaptive response with an improvement in the antioxidant defense system. Furthermore, there is an associated change in the redox balance (balance between oxidation and reduction) that produces a reducing environment, resulting in an increase in antioxidant defenses. This mechanism results in an adaptive protection against ROS during subsequent exercise sessions; for example, glutathione, a non-protein thiol, is produced and released [43].

GSH plays a central role in the maintenance of the reducing cellular environment, which is required to maintain the redox homeostasis, counteracting doxidative forces, and strengthening the antioxidant system [54–56]. However, inflammatory mediators and toxic substances can decrease the pools of GSH and other thiols [57]. Kooter et al. (2010), for example, observed that after an acute DEP exposure for 2 hours (1.9 μg/m^3^), the glutathione level was significantly decreased in the lung [58]. Our findings corroborate these results because the mice exposed to DEP for 10 days had a decrease in the levels of GSH and NPSH in the lung, but high-intensity swimming prevented this thiol loss, suggesting its influence on redox balance.

Redox balance is also maintained by the action of antioxidant enzymes. One of these enzymes is catalase, which degrades hydrogen peroxide into water and oxygen. Several pathogens produce catalase for defense against attacks by hydrogen peroxide, a weapon commonly used by the immune system of the host [59]. Some studies show that regular exercise may increase the levels of catalase, reestablishing organic homeostasis by increasing antioxidant defenses [60,61]. Corroborating those studies, our results showed that swimming resulted in an increase in the CAT activity levels in the lungs of mice, regardless of exposure to DEPs. Our results reinforce the idea that exercise, *per se*, can contribute to the enhancement of antioxidant defenses.

The increase in the oxidative stress induced by the exposure to toxic particulates, such as DEPs, is also associated with the release of pro-inflammatory cytokines in both clinical trials and experimental mice models [15,17,19,62,63].

DEP-induced ROS formation may activate transcription factors, such as NF-Kβ, involved in the regulation and release of pro-inflammatory cytokines [64,65]. Some studies suggest that pro-inflammatory reactions tend to start with the release of early responding cytokines, such as IL-1β and TNF-α, allowing an immediate response upon encounters with inhaled pathogens. IL-1β and TNF-α subsequently may regulate the expression of a variety of cytokines and chemokines, including IL-6. However, secondary cytokines may also be activated more directly by DEP in an IL-1β- and TNF-α-independent manner through activation of pro-inflammatory signaling pathways within the cells [22,66].

In this study, we showed that the lung IL-1β, TNF-α, IL-6 and IFN-ϫ levels were increased in mice that received DEP, corroborating the data of previous experimental studies [6,11,39]. Nevertheless, our results showed that high-intensity swimming resulted in a decrease in the levels of these pro-inflammatory cytokines.

In our experimental model, the lung levels of regulatory cytokines, such as IL-10 and IL-1ra in the mouse group subjected to swimming showed an increase, which was maintained in the DEP+Sw group, but it did not show significant difference when compared to DEP group. These results may be related to several features of the experimental design, including the activation time and the molecular pathways of the regulatory cytokines.

For instance, Yssel et al. (1992) showed that IL-10 levels were detectable in blood 8 hours after activation, and its maximal levels could be observed at 24 hours [67]. Furthermore, some studies also showed that the IL-10 and IFN-ϫ signaling pathways may share either common receptor subunits or intracellular activation pathways; thus, these molecules compete for binding sites [68]. In addition, the proposed interruption of the swimming sessions simultaneously with the addition of 5 more days of DEP administration in this experimental model could have influenced the IL-10 and IL-1ra results. The type, duration and intensity of exercise may be factors that influence the profile of the cytokine response [69].

Therefore, we conclude that in this experimental model, high-intensity swimming presented protective effects against DEP-induced lung inflammation, and these effects seems to be, at least partially, mediated by the antioxidant effects of exercise.
